# Know Your Noodles! Assessing Variations in Sodium Content of Instant Noodles across Countries

**DOI:** 10.3390/nu9060612

**Published:** 2017-06-16

**Authors:** Clare Farrand, Karen Charlton, Michelle Crino, Joseph Santos, Rodrigo Rodriguez-Fernandez, Cliona Ni Mhurchu, Jacqui Webster

**Affiliations:** 1 The George Institute for Global Health, The University of New South Wales, P.O. Box M20 Missenden Rd, Sydney 2006, Australia; mcrino@georgeinstitute.org.au (M.C.); jsantos@georgeinstitute.org.au (J.S.); jwebster@georgeinstitute.org.au (J.W.); 2 School of Medicine, Faculty of Science, Medicine and Health, University of Wollongong, Wollongong 2522, Australia; karen_charlton@uow.edu.au; 3 Illawarra Health and Medical Research Institute, Building 32, University of Wollongong Campus, Wollongong 2522, Australia; 4 Non-Communicable Diseases, International SOS, NCD Asia Pacific Alliance, Chiswick Park, 566 Chiswick High Rd, Chiswick, London W4 5YE, UK; rod.rodriguez@ncdapa.org; 5 National Institute for Health Innovation, University of Auckland, Private Bag 92019, Auckland Mail Centre, Auckland 1142, New Zealand; c.nimhurchu@auckland.ac.nz

**Keywords:** salt, sodium, salt reduction, ultra-processed food, instant noodles, blood pressure, non-communicable disease (NCDs), burden of disease, nutrition transition, regulation, salt targets

## Abstract

Reducing salt intake is a cost-effective public health intervention to reduce the global burden of non-communicable disease (NCDs). Ultra-processed foods contribute ~80% of dietary salt in high income countries, and are becoming prominent in low-middle income countries. Instant noodle consumption is particularly high in the Asia Pacific region. The aim of this study was to compare the sodium content of instant noodles sold worldwide to identify potential for reformulation. Analysis was undertaken for 765 instant noodle products from 10 countries using packaged food composition databases of ultra-processed foods compiled by the Global Food Monitoring Group (GFMG) and national shop survey data. Sodium levels were high and variable, within and between countries. Instant noodles in China had the highest mean sodium content (1944 mg/100 g; range: 397–3678/100 g) compared to New Zealand (798 mg/100 g; range: 249–2380 mg/100 g). Average pack size ranged from 57 g (Costa Rica) to 98 g (China). The average packet contributed 35% to 95% of the World Health Organization recommended daily salt intake of <5 g. Forty-one percent of products met the Pacific Island (PICs) regional sodium targets, 37% met the South Africa 2016 targets, and 62% met the UK 2017 targets. This study emphasises a need for stronger regulation and closer monitoring to drive rigorous reformulation of salt in ultra-processed foods.

## 1. Introduction

Cardiovascular disease (CVD) is the number one cause of death worldwide [1], responsible for 17.5 million deaths in 2012. High salt intake raises blood pressure, a major risk factor for CVD. Reducing population salt intake is recognised as a “best buy” for prevention and control of non-communicable diseases (NCDs) by lowering blood pressure and reducing risk of strokes and heart disease [2]. Salt reduction is considered a priority intervention by the World Health Organization (WHO) due to its high feasibility and potential to benefit to the whole population. Many countries are working towards achieving the global target of a 30% relative reduction in mean population salt intake by 2025, towards the WHO recommendation of <5 g/day [3].

Salt is a cheap food ingredient and ubiquitous in the food supply [4]. Salt is added to food products for the purposes of taste and preservation, and to improve technological processes [5]. In most high income countries, the majority of salt in the diet is from ultra-processed foods [6]. Thus, reformulation efforts to reduce the amount of salt added to ultra-processed foods are paramount to reduce population level salt intake. In general, in many low-middle income countries, the major source of salt in the diet is table salt and condiments added during cooking or at the table [6]. However, these countries are increasingly undergoing urbanisation and are experiencing a nutrition transition that is characterised by a marked change in food consumption patterns and a notable shift towards consumption of more ultra-processed foods [7,8]. 

A key example of this is instant noodles; an ultra-processed ultra-processed food product which is widely available at a low cost [9]. According to the World Instant Noodles Association (WINA), 270 million servings of instant noodles are consumed worldwide each day, with 80% of total consumption in Asian countries [10]. Instant noodles are consumed in more than 80 countries worldwide; China has the highest consumption of instant noodles, followed by Indonesia, Japan, and Vietnam [10]. 

In many Asian countries, noodles have been a staple food for centuries. Instant noodles are made from wheat flour, starch, water, salt, or kansui (an alkaline mixture of sodium carbonate, potassium carbonate and sodium phosphate), and other ingredients are added to improve the texture and flavour of the noodles [11]. Convenience, prolonged shelf life, taste, and low price make noodles highly popular. They can be eaten as a snack, as a meal or part of a meal, and some people consume them more than once a day. 

According to Fu [12], salt is used in the production of instant noodles at concentrations of 1–3% of flour weight, for the purpose of strengthening and tightening the gluten protein of the dough. Salt also serves to reduce cooking time, enhance flavour, provide a softer and more elastic texture, and inhibit enzyme activities and growth of microorganisms [12]. In addition, salt is a major component of the seasoning sachet that is generally included in the packaging of instant noodles and added at the time of consumption. 

Despite the widespread consumption of instant noodles in many countries, there has been relatively little assessment of their impact related to total nutritional intake and health. Analysis of dietary data collected in the Korean National Health and Nutrition Examination Survey (KNHANES) III, 2005, identified that consumers of instant noodles, compared to people who did not consume instant noodles, had significantly higher intakes of energy, fat, sodium, thiamine, and riboflavin and lower intakes of protein, calcium, phosphorus, iron, potassium, vitamin A, niacin, and vitamin C [13]. Analysis from KNHANES IV (2007–2009) demonstrated that the consumption of instant noodles two or more times per week was associated with a higher prevalence of metabolic syndrome in women (OR: 1.68; 95% CI: 1.10, 2.55) and that this association was independent of major dietary patterns [14]. 

Given emerging data of this kind, reformulation of instant noodles is important to reduce their potentially harmful nutritional composition. Programs to engage with the food industry have been undertaken by many countries worldwide, with some countries already, reporting an impact [15]. However, few countries have set targets specifically for instant noodles to date. The United Kingdom (UK) has set an average target of 200 mg of sodium and a maximum of 350 mg of sodium per 100 g of instant noodles “as prepared” (made up according to manufacturer instructions) [16]. South Africa (SA) set legislative targets of 1500 mg of sodium/100 g by 2016 and of 800 mg of sodium per 100 g by 2019 “as sold” [17], similar to the PICs regional target of 1600 mg/100 [18]. “As sold” refers to 100 g of product before it is made up with water, ready to eat.

Through the Global Food Monitoring Group (GFMG) [19], the George Institute for Global Health has been supporting countries to establish comprehensive food composition databases (FCDs) to monitor the nutritional composition of packaged food, which can be used to drive national and international improvements to the food supply, and improve the health of billions of people worldwide. The aim of this analysis was to assess sodium levels in instant noodles using data from the GFMG national databases as well as from countries that have recently collected sodium data for instant noodles as part of shop surveys. The mean values and ranges of sodium content of instant noodles were compared, both within and between countries, and sodium content was compared against existing sodium targets for instant noodles [16,17,18]. The purpose of the analysis was to compare the sodium content of instant noodles sold worldwide to monitor sodium levels against existing targets and to identify opportunities to reformulate instant noodles as a means to reduce population level salt consumption. 

## 2. Materials and Methods 

Data on instant noodles collected between 2012 and 2016 were extracted from existing packaged food composition databases from countries that are part of the Global Food Monitoring Group (GFMG) [19]. Data on instant noodles were also gathered from countries that have recently been supported by the George Institute to gather shop survey data as part of surveillance activities to monitor sodium contents of the food supply (Table 1). All data were collected systematically by trained research assistants in accordance with the GFMG protocol. Data from the respective databases included brand name, product name, pack size, serving size, sodium mg/100 g “as sold”, sodium mg/100 g “as prepared”, salt g/100 g “as sold”, and salt g/100 g “as prepared”.

### 2.1. Data Categorisation 

Instant noodles were defined according to Codex Alimentarius [20], as packaged noodles, with or without additional seasonings provided in separate pouches, ready for consumption after rehydration. Data was categorised into two main groups: “as sold” or “as prepared” according to the listed nutrition information. Products categorised “as sold” listed sodium information based on dry weight including the seasoning. Products that were categorised “as prepared” listed sodium information based on the product as prepared for consumption according to manufacturer instructions, for example, “add x millilitres of water” and included an addition of the seasoning in the sodium value.

### 2.2. Data Analysis

The total number of instant noodle products and the number of products with sodium or salt information was recorded for each country. Sodium content was calculated from salt where salt information alone was provided on the packaging, using the conversion factor of Na (mg) = salt (mg) (NaCl)/2.5. The mean, median, and ranges of sodium (mg/100 g) were calculated for each category (“as sold” and “as prepared”) for each country. Average pack size, “as sold”, and average portion size, “as prepared”, were derived from available data as given on pack for each country. Mean sodium values of instant noodle products reporting sodium “as sold” were compared against the SA and PICS regional targets, while those reporting sodium “as prepared” were compared against the UK 2017 targets. The salt targets were converted to sodium for ease of comparison where necessary. The proportion of products known to meet the sodium targets were derived for each country. The contribution of an average packet of instant noodles to the WHO’s recommended intake of <2000 mg of sodium (5 g salt) per day was derived using mean sodium values and average pack size for each country.

Sub-analyses comparing sodium content of noodles of countries by income level (based on The World Bank’s list of economies [21]) and by whether or not they had specific sodium targets in place for instant noodles were conducted. Median sodium content (mg/100) was compared using the Wilcoxon rank-sum test. The proportion of products meeting the target between groups was compared using the chi-square test. A *p*-value of < 0.05 was considered significant. 

## 3. Results

Data were collated on 765 instant noodles products from 10 countries. China had the greatest number of noodle products (283 products, 37% total), followed by the UK (137, 18%), New Zealand (85, 11%), and Australia (58, 8%). Indonesia and Costa Rica had the fewest products, 28 and 18 products respectively (Table 1).

### 3.1. Labelling

Five percent of products did not list sodium or salt content on the nutrition information panel; the majority of these were from India. Sixty-eight percent of instant noodle products in India did not provide sodium content information. Of all the products that did display sodium information, approximately 67% of products listed nutrition information “as sold”, while 33% listed nutrition information “as prepared” (Table 1). Most (92%) of the noodle products from the UK listed nutrition information “as prepared”, compared to China, Indonesia, India, and Costa Rica, which all listed the nutrition information “as sold”. In Australia and New Zealand, 84% and 49%, respectively, of noodle products listed their nutritional content “as prepared”.

### 3.2. Range and Levels of Sodium Per 100 g

There was a wide range in sodium content of instant noodles within and between countries, and the distribution of sodium was not normal, so median sodium values were also reported (Table 2). The highest mean and median sodium content (mg/100 g) of instant noodles “as sold” was found in products in China (mean 1944, median 2062, IQR 757, range 397–3678) followed by Australia, Fiji, Samoa, and Indonesia. The lowest mean sodium content (mg/100 g) was found in products in New Zealand (mean 798, median 508, IQR 429, range 249–2380).

The highest mean sodium content (mg/100 g) of instant noodles “as prepared” was for products in New Zealand (mean 388, median 360, IQR 106, range 222–725), whilst the lowest mean sodium content (mg/100 g) “as prepared” was for products in the UK (mean 220, median 200, IQR 100, range 120–440).

### 3.3. Percentage of Products which Meet Targets

Forty percent of all products met the PICs targets for instant noodles (1600 mg/100 g “as sold”); 37% met SA 2016 targets (1500 mg/100 g “as sold”) and 72% met the UK 2017 maximum target (350 mg of sodium/100 g “as prepared”). Among countries with targets, 26% and 39% of instant noodles in Fiji and Samoa, respectively, met the PICs regional salt targets; 86% of products in SA met the SA salt targets, and 90% of products in the UK met the UK 2017 salt targets.

Sub-analysis comparing countries with sodium targets (either “as sold” or “as prepared”) against countries without targets in place showed that countries with targets for instant noodles had a significantly higher proportion of instant noodle products meeting the targets. The proportion of products meeting the PICs, SA, and UK targets in countries with salt targets for instant noodles compared to those without targets were 56% vs. 36% (*p* = 0.001), 47% vs. 34% (*p* = 0.031), and 86% vs. 49% (*p* < 0.001), respectively.

### 3.4. Average Pack Size

**Figure 1:**
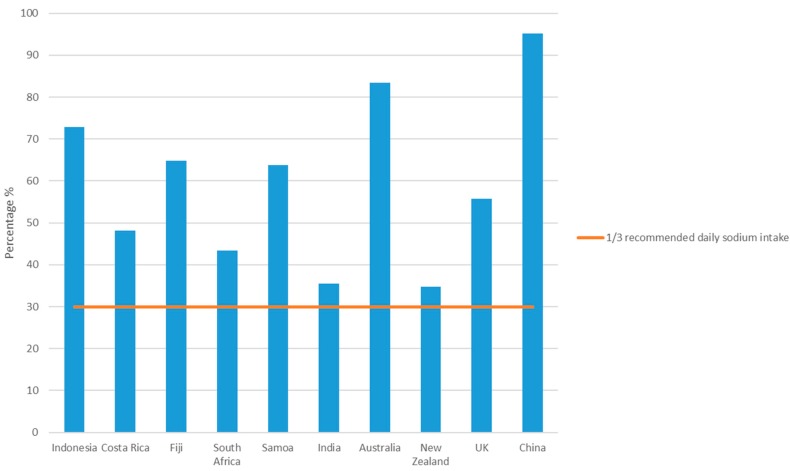
Estimated sodium contribution (%) of an average packet of instant noodles “as sold” with maximum recommended daily sodium intake (2000 mg/day).

There were large variations in both pack sizes and serving sizes. Average pack size ranged from 57 g in Costa Rica to 98 g in China. Where serving size information was given, average serving size, “as prepared”, ranged from 143 g in Fiji to 300 g in South Africa. Six out of 10 countries provided serving size information on the instant noodles packaging (Table 1). Based on an average packet of noodles “as sold”, the estimated average contribution of one packet of noodles towards the World Health Organization daily recommended maximum intake of sodium (<2000 mg) ranged from 35% in India and New Zealand (628 mg per pack and 697 mg per pack, respectively) to 95% in China (1905 mg per packet in China) (Figure 1).

### 3.5. Comparison of Sodium Levels in Instant Noodles between High-Income Countries and Middle-Income Countries 

Seven of the 10 countries included in the analysis were classified as middle-income countries (MICs), and 3 classified as high-income countries (HICs); there was no data from low-income countries. Median sodium level of instant noodles “as sold” were significantly higher in MICs (1889 mg/100 g) compared to HICs (605 mg/100 g) (*p* < 0.001). In addition, a significantly higher proportion of products in HICs compared to MICs met the Pacific Island (71% vs. 35%, *p* < 0.001) and South Africa salt targets (71% vs. 32%, *p* < 0.001). There was no significant difference for instant noodles with mean sodium levels “as prepared”.

## 4. Discussion

This assessment of the sodium content of 765 instant noodles products from 10 countries demonstrated extremely wide variation in sodium content, both between and within countries, according to product ranges and brands. The huge variations in mean sodium content of products in different countries clearly demonstrates significant potential to reduce the sodium content of noodles sold worldwide.

Reasons for the wide range of sodium content of instant noodles between countries cannot be explained solely by taste preferences of consumers, as there were also vast differences in the sodium levels of the instant noodles on the market within each country. For example, the sodium content of instant noodles in Australia ranged from 950 mg/100 g to 3050 mg/100 g (“as sold”), with the highest sodium instant noodle product containing over 3 times more sodium than the lowest sodium instant noodle product. This shows clearly that manufacturers are able to produce instant noodles with far less sodium and that these products are already accepted by consumers. This is evidence that reformulation of instant noodles is feasible, both technologically as well as from a consumer acceptability perspective. Similar results were observed looking at the analyses of median values, which confirms the main findings.

Whilst recognising that the targets only apply to the countries or regions in which they were set, the fact that the sodium content of instant noodles was consistently lower in countries with targets demonstrates the effectiveness of targets as a public policy tool. For example, China, which has the highest number of instant noodle products, does not have targets for sodium levels in foods, and had the lowest proportion of instant noodle products meeting international targets. However, there is scope for greater compliance, as not all products within countries with targets, met the targets, which points toward the need for more concerted efforts to reduce salt by the food industry. This highlights the importance of monitoring frameworks to allow for the transparent and objective evaluation of the food industry towards meeting the targets. In Fiji and Samoa for example, only 39% and 26% of products, respectively, met the PICS regional targets. The targets are part of a voluntary framework in Fiji and are being incorporated into regulations that have yet to be implemented in Samoa. Most Pacific Island countries are also highly dependent on imports, which means they need to work with food importing companies as well as local manufacturers to implement targets and highlights the importance of strong government leadership to support policy implementation. The high level of sodium in instant noodles coupled with their popularity provides a strong case for sodium reduction targets for instant noodles in all countries.

Both voluntary and legislated sodium reduction targets can lead to industry action to reduce sodium; 90% of instant noodle products in the UK met the UK targets (which were voluntary targets to be achieved by 2017), and 86% of products in South Africa met the South African mandatory targets to be achieved by 2016. Public health experts believe that regulation is a much stronger driver for industry reformulation [22], but voluntary programs that are supported by a strong monitoring framework, coupled with strong advocacy efforts, are also making considerable progress [23]. 

This research also identified that instant noodles in middle-income countries have a significantly higher average sodium content compared to high-income countries. This is of concern, given that nutrition transition is resulting in an increased availability of more ultra-processed foods in these countries [24], and supports the need for robust policies to regulate the food supply to reduce the already overburdened constraints on the healthcare system due to poor diet. 

Further to the need for reformulation of instant noodles to contain less sodium, this study highlights the need for clear and consistent nutrition information panels (NIPs) to enable consumers to make healthier food choices. In India, 68% of products did not list sodium data on nutrition information panels, thus failing to meet International Codex Alimentarius requirements [25]. The Food Standards and Safety Authority of India does not currently require reporting of sodium content on food packaging [26]. This lack of nutrition information not only inhibits consumers from making informed choices about food purchases but also prevents any monitoring and evaluation of the food supply.

The fact that some instant noodle manufacturers label sodium information on nutrition information panels “as sold” while others label sodium “as prepared” (as prepared according to manufacturer instructions) within the same country further complicates the picture for both consumers and policy makers. In New Zealand, for example, almost half of the products labelled sodium information “as sold” and half “as prepared”. This creates potential confusion for consumers at the point of purchase and makes it difficult to compare nutrition information between brands. Almost a third of all instant noodle products analysed labelled sodium information “as prepared” according to manufacturer instructions. Consumers may not necessarily follow manufacturers’ instructions in preparation of the product, which introduces further potential bias in estimates and makes it more difficult for consumers to moderate salt intakes. In countries with interpretive front of pack labelling, for example, the colour coded Front of pack (FOP) nutrition labelling scheme in the UK [27], there is an opportunity for these systems to signpost healthier alternatives and make direct comparisons between products easier for consumers at the point of purchase.

Inconsistent pack sizes provide an additional challenge; the results from this survey showed that pack sizes ranged from 57 g in Costa Rica to 97 g in China and that manufacturers did not always quantify a recommended serve size. A single packet of one brand of instant noodles in China contributes almost the entire (95%) WHO recommended maximum <2000 mg of sodium/day. In Indonesia, Fiji, or Samoa, the average packet of noodles would contribute almost two-thirds of this amount, whereas in India and New Zealand consumers would consume almost one-third. 

Ensuring that instant noodles are reformulated to reduce sodium and labelled in a meaningful way is even more important given the fact that they are increasingly being promoted as a vehicle for micronutrient fortification, either added through the flour used to make the product or to the seasoning powders consumed with the noodles [28,29]. This practice may result in contradictory public health outcomes.

There were some limitations to the study. The number of products available in the ultra-processed packaged food composition database may not necessarily reflect the number of products sold in a particular country, but rather those that were captured during shop surveys undertaken within a limited subset of retail outlets, at specific time points. Products were categorised “as sold” or “as prepared” according to the investigators’ best interpretation, using mean sodium as a guide. Products that could not be categorised were excluded from further analysis. Products were collected in shop surveys between 2012 and 2016 and may no longer be on sale due to stock changes, and sales data could not be corroborated to assess market share of particular brands. However, the data obtained provides a clear indication of the high levels of sodium in these popular products, which supports the need for reformulation efforts worldwide to reduce sodium in instant noodles to the lowest possible level. 

## 5. Conclusions

The high level of sodium in instant noodles across the world is a major public health concern, given their low cost, convenience, and widespread availability, and the fact that high sodium levels are a key contributor to ill health. There is a need for clear targets coupled with rigorous reformulation efforts to reduce the amount of sodium added to instant noodles. Better regulation of the sodium content of commonly consumed ultra-processed foods is key to reducing population-level salt consumption around the world. 

## Figures and Tables

**Table 1:** Proportion of instant noodles collected per country and average pack/serving size (g).

Date of Data Collection	Data Source	Country	Total No. of Products Collected	Products with Sodium Data		Products with Sodium Data “as Sold”		Products with Sodium Data “as Prepared”		Average Pack Size (g)	Average Serving Size (g) as Prepared
				*n*	%	*n*	%	*n*	%		
2016	FCD	UK	137	132	96	11	8	121	92	84	282
2015	FCD	New Zealand	85	83	98	42	51	41	49	87	343
2014	FCD	Australia	58	58	100	9	16	49	84	86	308
2015	FCD	China	283	283	100	283	100	0	0	98	-
2012	FCD	India	47	15	32	15	100	0	0	-	-
2013	Shop survey	Samoa	44	43	98	28	65	15	35	69	-
2015	FCD	South Africa	37	37	100	28	76	9	24	72	300
2013	Shop survey	Fiji	28	28	100	23	82	5	18	69	143
2015	Shop survey	Indonesia	28	28	100	28	100	0	0	76	-
2013	FCD	Costa Rica	18	18	100	18	100	0	0	57	-
Totals			765	725 *	95	485	67	240	33	78	275

- data not available.* Products that listed sodium or salt information, but did not state if as sold or as prepared were excluded from further analysis (*n* = 5).

**Table 2:** Mean, range, median interquartile range, and percentage of products that meet sodium targets.

Country (World Bank Group)	Products with Sodium Data “as Sold”								Products with Sodium Data “as Prepared”					
	*n*	Mean Sodium (mg/100 g) as Sold	Range of Sodium (mg/100 g) as Sold	Median Sodium and IQR (mg/100 g) as Sold	Products Known to Meet Pacific Salt Reduction Target (1600 mg/100 g as Sold)		Products Known to Meet South Africa 2016 Target (1500 mg/100 g as Sold)		*n*	Mean Sodium (mg/100 g) as Prepared	Range (mg/100 g) as Prepared	Median Sodium and IQR (mg/100 g) as Prepared	Products Known to Meet UK 2017 Max Sodium Target (350 mg/100 g as Consumed)	
					*n*	%	*n*	%					*n*	%
New Zealand (HIC)	42	798	249–2380	508 (429)	35	83	35	83	41	388	222–725	360 (106)	19	46
UK (HIC)	11	1323	488–2650	948 (1620)	6	55	6	55	121	220	120–440	200 (100)	109	90
Australia (HIC)	9	1939	950–3050	2110 (951)	3	33	3	33	49	378	205–635	350 (181)	25	51
China (MIC)	283	1944	397–3678	2062 (757)	74	26	67	24	0	-	-	-	-	-
Samoa (MIC)	28	1854	970–3360	1751 (610)	11	39	6	21	15	334	245–590	280 (75)	12	80
Indonesia (MIC)	28	1916	770–7584	1388 (1025)	15	54	15	54	0	-	-	-	-	-
South Africa (MIC)	28	1206	350–1640	1314 (202)	27	96	24	86	9	331	266–475	290 (90)	5	56
Fiji (MIC)	23	1892	845–3510	1913 (767)	6	26	6	26	5	317	200–443	300 (184)	3	60
Costa Rica (MIC)	18	1703	1148–2278	1766 (242)	4	22	4	22	0	-	-	-	-	-
India (MIC)	15	910	280–1932	590 (1067)	12	80	12	80	0	-	-	-	-	-
HICs sub-total	62	1057	249–3050	605 (1280) *	44	71 *	44	71 *	211	289	120–725	270 (180)	153	73
MICs subtotal	423	1838	280–7584	1889 (926)	149	35	134	32	29	330	200–590	290 (98)	20	69
Totals	485	1738	249–7584	1823 (1029)	193	40	178	37	240	294	120–725	273 (160)	173	72

* Difference between HICs and MICs significant at *p* < 0.001. - data not available.
